# Lactated Ringer’s Solution Reduces Severity, Mortality, Systemic and Local Complications in Acute Pancreatitis: A Systematic Review and Meta-Analysis

**DOI:** 10.3390/biomedicines11020321

**Published:** 2023-01-23

**Authors:** Klementina Ocskay, Péter Mátrai, Péter Hegyi, Andrea Párniczky

**Affiliations:** 1 Heim Pál National Pediatric Institute, 1089 Budapest, Hungary; 2 Institute for Translational Medicine, Medical School, University of Pécs, 7623 Pécs, Hungary; 3 Centre for Translational Medicine, Semmelweis University, 1085 Budapest, Hungary; 4 Institute of Pancreatic Diseases, Semmelweis University, 1083 Budapest, Hungary; 5 Translational Pancreatology Research Group, Interdisciplinary Centre of Excellence for Research Development and Innovation, University of Szeged, 6725 Szeged, Hungary

**Keywords:** acute pancreatitis, fluid therapy, resuscitation, crystalloid

## Abstract

Fluid therapy is the cornerstone of early supportive therapy in acute pancreatitis (AP). Regrettably, the type of fluid is still debated among clinicians, despite recent evidence from randomized controlled trials (RCTs). We aimed to incorporate all evidence from RCTs comparing lactated Ringer’s solution (LR) with normal saline (NS) in adult and pediatric AP patients, with particular emphasis on clinically relevant outcomes. We evaluated RCTs comparing intravenous fluid resuscitation with LR to NS in adult or pediatric AP patients according to a prospectively registered protocol (CRD42021224542). Moderate-to-severe AP (MSAP), mortality, length of hospitalization (LoH), need for intensive care, the incidence of systemic (organ failure, OF) and local complications (in total), necrosis and pseudocyst formation were analyzed separately. Risk ratio (RR) and median difference (MD) were calculated with 95% confidence intervals (CI) using a random effect model. Risk of bias and quality of evidence were assessed. Altogether, 8 eligible RCTs were found, including 557 patients (LR: 278; NS: 279). LR reduced the risk of MSAP by 31% (RR: 0.59, 95% CI: 0.36–0.97, high quality) and the risk of death by 62% (RR: 0.48; 95% CI: 0.24–0.98, very low quality). LR was associated with a significantly lower risk of need for intensive care (RR: 0.50, 95% CI: 0.33–0.77), OF (RR: 0.78, 95% CI: 0.61–0.99) and local complications (RR: 0.64, 95% CI: 0.46–0.89). No significant risk reduction was observed for LoH (MD: −0.57 days, CI: −1.33–0.19), necrosis, pseudocyst and inflammatory parameters by LR compared to NS. LR reduces severity, mortality, need of intensive care and systemic and local complications in AP.

## 1. Introduction

The significance of acute pancreatitis (AP) lies in its relatively high incidence, rising prevalence and the economic burden it poses on society [1,2,3,4]. The general consensus on diagnostic criteria and severity assessment resulted in high-quality research and recent therapeutic advances [5]. To date, no specific therapy exists, except the elimination of the etiological factor [6,7,8], but it is clear that curbing systemic inflammation results in better outcome [9]. This underlines the importance of initial management.

It is widely acknowledged that fluid therapy is the cornerstone of early supportive therapy for AP. Nonetheless, the amount and type of fluid is still debated among clinicians, despite recent evidence from randomized controlled trials (RCTs) [10,11]. Albeit initial results were encouraging [12,13], the use of colloids as routine resuscitation fluid was proven to be harmful both in critically ill patients [14,15] and in severe acute pancreatitis [16]. Still, the best choice among available crystalloids was not established. Lactated Ringer’s solution (LR) came into focus after Wu et al. conducted an RCT using LR for goal-directed fluid therapy in AP [17]. They hypothesized that a balanced crystalloid may have advantages over normal saline (NS), since acidosis can contribute to the worsening of AP [18,19]. In a study published by Hoque et al., in vitro experiments confirmed the immunomodulatory effects of lactate [20]. Based on these results, several investigator-initiated RCTs were published between 2018 and 2022 around the world.

The worldwide effort of individual researchers and our experience shows [21,22] that physicians involved in pancreatitis care need further affirmation on the superiority of LR to NS. Several meta-analyses attempted to summarize the currently available evidence [16,23,24,25,26,27,28,29]. As multiple trials were published after the last search was conducted for these systematic reviews [30,31], a more comprehensive analysis could be decisive in this question.

We aimed to incorporate all the evidence from RCTs comparing LR with NS in adult and pediatric AP patients in a meta-analysis, with particular emphasis on clinically important outcomes, including mortality, severity and local and systemic complications.

## 2. Materials and Methods

### 2.1. Protocol, Registration and Reporting

The protocol was prospectively registered on PROSPERO (CRD42021224542). Data were mainly reported as median and interquartile; therefore, we used median difference instead of mean difference, as previously stated. We followed the recommendations of the ‘Preferred Reporting Items for Systematic Reviews and Meta-Analyses (PRISMA)’ group [32].

### 2.2. Eligibility Criteria

RCTs, regardless of the participants’ age and length of follow-up, reporting on the effects of LR compared to NS in AP were considered eligible. Non-randomized trials and cohort studies and studies addressing the prevention of AP were excluded. We assessed the following outcomes: severity; mortality; length of hospital stay (LoH); organ failure (defined by the Atlanta classification as systemic complication, OF); local complications, including necrosis and pseudocyst formation; need for intensive care unit (ICU) admission; systemic inflammatory response syndrome (SIRS); C-reactive protein (CRP; mg/dL) level; and, additionally, the amount of fluid administered.

### 2.3. Systematic Search, Selection and Data Extraction

MEDLINE (via PubMed), EMBASE, Scopus, Web of Science and Cochrane Central Register of Controlled Trials (CENTRAL) were searched until 2nd of September 2022 using the following search key: (acute pancreatitis) AND (ringer* OR hartmann*). Only the title, abstract and keywords were searched in the Scopus database. No other filters or restrictions were applied. Citations were exported to a citation manager software (EndNote X9, Clarivate Analytics, Philadelphia, PA, USA). The selection was conducted following the recommendations of the PRISMA 2020 Statement, by two independent review authors (K.O. and A.P.) using a predetermined set of rules. Disagreements were settled by consensus. The rate of agreement was determined by calculating Cohen’s kappa coefficient [33] (k = 0.96 for title, k = 0.96 for abstract and k = 1.0 for full text).

Prespecified pieces of information were extracted to a Microsoft Excel sheet by K.O. and validated by A.P.

### 2.4. Risk of Bias Assessment

The Revised Risk of Bias Assessment Tool (RoB 2) was used. Detailed results are presented in Figure S1 (references in Table S2).

### 2.5. Statistical Analysis

Analyses were made with R [34], using the meta [35,36] and metamedian [37] packages.

For binary outcomes, the risk ratio (RR) with 95% confidence interval (CI) was used for the effect measure. To calculate the RR, the total number of patients in each group and those with the event of interest were extracted from each study. Raw data from the selected studies were pooled using a random effect model with the Mantel–Haenszel method. For the pooled results, the exact Mantel–Haenszel method (no continuity correction) was used to handle zero cell counts [38]. In case of continuous outcomes, the median difference (MD) with 95% CI was calculated as effect size. The extracted values to estimate the MD and its variance were the sample size, the median, the lower and upper quartiles, and the minimum and maximum values in the two groups, if available. To estimate the median and its variance in studies reporting mean and standard deviation, the distribution was assumed to be normal. The sampling variance of the medians was estimated by the QE method (“Meta-Analysis of the Difference of Medians” 2020), and the random effect model was used to summarize the median differences. The Hartung–Knapp adjustment was applied to avoid false positive findings [39,40].

To estimate τ^2^, we used the restricted maximum likelihood method. Statistical heterogeneity across trials was assessed by means of the Cochrane Q test and the I^2^ values [41].

Forest plots were used to graphically summarize the results. Where applicable, we reported the prediction interval (i.e., the expected range of effects of future studies), following the recommendations of IntHout et al. [40]. Due to the low number of studies, publication bias was not evaluated. For all outcomes, statistical significance was defined as *p*-value <0.05.

### 2.6. Determination of Quality of Evidence

We used the ‘Grading of Recommendations Assessment, Development and Evaluation’ (GRADE) working group’s recommendation for assessing the quality of the evidence. Detailed results are presented in Table S1.

## 3. Results

### 3.1. Study Selection

**Figure 1:**
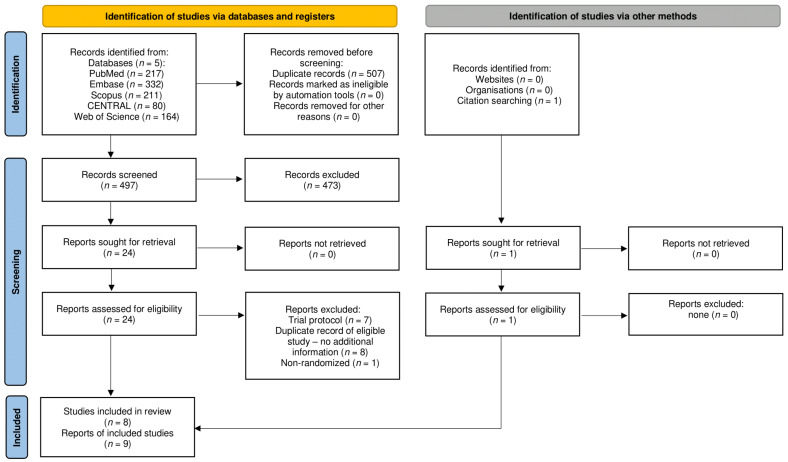
Flow diagram of study selection process.

Altogether, 1004 records were identified by the systematic search, and 1 additional record was found during the overview of the references and citations of eligible studies and the meta-analyses of the same topic [42]. After the selection process shown in Figure 1, eight eligible studies were identified, reported on by six full texts [17,30,31,43,44,45] and three conference abstracts [42,46,47]. Abstracts by Vasudevan and Reddy are considered multiple reports of the same RCT.

The main characteristics of the included studies are depicted in Table 1.

### 3.2. Study Characteristics

The main characteristics of the included studies are collected in Table 1. Altogether, 557 patients were enrolled in the 8 RCTs we found eligible. Seven out of the eight studies were conducted on adult patients. The study by Farrell et al. enrolled exclusively pediatric patients with a mean age of 12 years. Two multicenter studies were included in our analysis, both of which were conducted in the United States of America [17,47]. AP was defined using the Atlanta Classification in the majority of the studies, while Wu and Reddy/Vasudevan et al. did not provide information on diagnostic criteria. Most studies used goal-directed fluid protocols, with an initial bolus and continuous fluid administration until toleration of an oral diet. Only two studies reported on fluid administration prior to randomization [17,45]. Reported outcomes are listed in Table 1.

### 3.3. Quantitative Synthesis

Main findings for all outcomes included in the meta-analyses are summarized in Table 2.

### 3.4. Severity and Mortality of Acute Pancreatitis

Across three studies, lactated Ringer’s solution (LR) reduced the risk of MSAP by 31% (RR: 0.59, 95% CI: 0.36–0.97, *p* = 0.045; I^2^: 0%, *p* = 0.692; high quality, Table 2, Figure S2). Farrell et al. reported a similar incidence of severe AP in both groups (11% in LR, 5% in NS, *p* = 0.67) [47]. Reddy et al. also did not find a difference in severity (*p* = 0.77) [42]. Additionally, Kayhan et al. assessed severity with the Modified CT Severity Index besides the Atlanta Classification, which was similar in the two groups (*p* = 0.238) [31].

Five studies reported on in-hospital mortality, but only three patients died in the whole study population. LR fluid therapy was associated with a significantly lower risk of death compared to NS (RR: 0.48; 95% CI: 0.24–0.98, *p* = 0.047; I^2^: 0%, *p* = 0.979; very low quality, Table 2, Figure S3). Additionally, Choosakul et al. provided data for 30-day mortality, which were identical to in-hospital mortality [43].

### 3.5. Length of Hospitalization and Need for Intensive Care

Patients infused with LR and NS had similar LoH (MD: −0.57, 95% CI: −1.33–0.19, *p* = 0.120; I^2^: 35.7%, *p* = 0.100; moderate quality, Table 2, Figure S4). Data were available for eight studies, including the only pediatric study, published by Farrell et al. [47]. They reported the number of patients discharged at 48 and 72 h and found that patients from the LR group were discharged sooner. Lee et al. also reported the 72-hour discharge rate, which was higher among patients infused with LR (44% vs. 28.3%) [45].

Nevertheless, across four studies, LR fluid therapy significantly reduced the need for intensive care by 50% (RR: 0.50, 95% CI: 0.33–0.77, *p* = 0.014; I^2^: 0%, *p* = 0.861; low quality, Table 2, Figure S5).

### 3.6. Complications

In the Revised Atlanta Classification, systemic complications of AP are defined as respiratory, cardiovascular or renal organ failure (OF). Therefore, data on the occurrence of OF were pooled. Across six studies, LR reduced the development of OF by 22% (RR: 0.78, 95% CI: 0.61–0.99, *p* = 0.046; I^2^: 0%, *p* = 0.842; low quality, Table 2, Figure S6).

Local complications were also assessed according to the Revised Atlanta Classification. LR fluid therapy reduced local complications by 36% (RR: 0.64, 95% CI: 0.46–0.89, *p* = 0.023; I^2^: 0%, *p* = 0.856; moderate quality, Table 2, Figure S7). Data were sufficient for the separate analysis of necrosis and pseudocyst, but did not permit the quantitative analysis of the risk of walled-off necrosis and peripancreatic fluid collection. No statistically significant association was found between LR fluid therapy and the development of necrosis (RR: 0.70, 95% CI: 0.40–1.23, *p* = 0.176; I^2^: 0%, *p* = 0.618; low quality, Table 2, Figure S8) or pseudocysts (RR: 0.78, 95% CI: 0.61–8.68, *p* = 0.950; I^2^: 0%, *p* = 0.659; low quality, Table 2, Figure S9) during the course of AP. The incidence of walled-off necrosis and peripancreatic fluid collection were reported by Choosakul and Karki et al. with no significant differences [30,43].

### 3.7. Systemic Inflammation

No statistically significant association was identified between LR and the development of SIRS at any time point (Table 2, Figure S10). Furthermore, across 3 studies, no association was found between LR and CRP levels at 48 h from initiation of fluid therapy (Table 2, Figure S11). A total of 2 studies reported CRP levels at 24 h and 1 at 72 h (Figure S11).

### 3.8. Additional Outcomes

We deemed it important to compare the amount of fluid administered in the study groups, as it could influence the main outcomes. Four studies reported the amount of fluid administered in each study group, and no statistically significant differences were found (Table 2, Figure S12).

Additional outcomes reported by the eligible studies are listed in Table 1.

## 4. Discussion

As no pharmacological therapy is available, currently the cornerstone of initial management is fluid therapy [5,48,49]. The rate and fluid type, however, are far from unambiguous for the average clinician. Recently, the results of the Waterfall trial were published, assessing aggressive fluid resuscitation in AP [11]. Early termination was initiated after the first interim analysis, as fluid overload—the main safety outcome—was significantly more frequent in the aggressive resuscitation group (20.5% vs. 6.3%). However, it must be noted that strict eligibility criteria resulted in a patient population without significant dehydration.

Regarding the type of resuscitation fluid reviews, expert opinion pieces and even recent guidelines highlight the potential benefit of LR in AP [10,50,51,52]. However, their reasoning may be insufficient to convince physicians to change an affordable, widely used crystalloid for another. 

Even though isotonic, normal saline is not a balanced crystalloid solution [53]. Caused by its different strong ion concentration to plasma, NS infusion may result in hyperchloremic acidosis and promote kidney injury [54]. In vitro data suggest that extracellular acidosis is a danger signal, resulting in the activation of innate immunity [55]. Acidosis in the context of AP was also described as a factor negatively influencing the outcome [18,19]. In contrast, LR is a balanced crystalloid, where lactate acts as a buffer to prevent acidosis. Lactate has been shown to moderate inflammation through the TLR4 pathway, negatively influencing NLRP3 inflammasome and interleukin-1β production [20]. Furthermore, extracellular calcium supplementation by LR infusion could also mitigate lipotoxic injury, preventing necrosis [56].

Although both LR and NS solutions were administered frequently in clinical settings for over a century, few clinical trials addressed the specific question of LR versus NS. Since 2018, evidence on the superiority of LR to NS in clinical settings is available from large RCTs conducted on both critically ill [54] and non-critically ill inpatients [57]. However, changes in everyday practice are gradual.

Leading researchers in the field may think it evident that the fluid of choice in the initial phase of AP is LR, but the slow translation of scientific results into clinical practice and delayed adoption of evidence-based practices are also observable in this matter [58,59]. A survey of 1054 physicians from 94 countries conducted in 2021 showed that still almost one third of doctors (31%) prefers NS or other solutions, but not LR [60]. Surprisingly, 29% of doctors actively managing AP patients preferred other solutions rather than LR according to this survey [60]. It is also of note that older physicians and doctors treating AP patients for at least 10 years tend to neglect the recommendations on LR use in AP.

Most of the articles reviewing evidence in AP highlight the positive effect of LR fluid therapy on CRP levels and SIRS. It is eminent that the pathophysiology of AP revolves around trypsinogen activation and the generalization and escalation of the inflammatory response, resulting in organ failures and adverse outcomes [5]. Since LR may have anti-inflammatory effects, based on in vitro and animal research [20,56], it was a sensible choice until clinical trials and meta-analyses provide firm evidence. All RCTs assessing the specific question in focus—and involved in our meta-analysis—were designed focusing on the mediation of systemic inflammation assessed by SIRS and CRP levels. Although our workgroup has previously shown that CRP levels are associated with severity in AP [21], information on clinically relevant outcomes, such as severity, mortality and complications, was needed to provide direct evidence on the benefits of LR in AP. The quality of evidence for our results reflects that the available RCTs were not designed for these outcomes.

Although several types of crystalloid solutions are used in clinical practice, except one observational study comparing PlasmaLyte and NS, no evidence is available. Iqbal et al. found that PlasmaLyte use was associated with significantly shorter LoH, fewer SIRS at 48 h and a lower 30-day readmission rate [61]. Based on these findings, the benefits demonstrated by LR and PlasmaLyte could be attributed to their balanced nature and lack of acidosis caused by hyperchloremia. However, it should be further investigated whether LR is superior to other types of balanced crystalloid solutions in AP. Otherwise, the current statements exclusively recommending LR for resuscitation in AP should be modified. In our opinion, this would promote the use of balanced crystalloids, rather than NS, in AP care and result in better outcomes generally.

### 4.1. Strengths and Limitations

We conducted a comprehensive meta-analysis with clinically relevant outcomes in focus on the benefit of LR fluid therapy, rather than NS, in AP. Compared to the previously published meta-analyses on this topic, an additional 3 RCTs were included in our analysis, totaling 8 studies with 557 patients. The scope of our review was not limited to adult patients, as fluid therapy in pediatric AP is also of critical importance. Besides previously assessed outcomes, we were able to analyze CRP levels and pseudocyst formation. As data were frequently published as median and quartiles, the use of median difference, rather than mean difference, decreases imprecision by limiting the use of estimated values.

Our study has several limitations, as well. Despite the increased number of studies, the optimal information size was not reached for several outcomes. This, and other limitations of the included studies—including the risk of bias—are reflected by the grade of evidence, which was low or very low in five out of eight assessed outcomes.

### 4.2. Implications

Translating scientific results to daily practice has crucial importance. LR should be the standard choice for initial fluid resuscitation in AP, using a goal-directed approach and close surveillance of volume status.

Further studies assessing fluid therapy in AP should be carried out in international collaboration to be adequately powered to elevate the level of evidence for specific outcomes if deemed necessary.

## 5. Conclusions

Lactated Ringer’s solution reduced severity, mortality, need for intensive care, organ failure and local complications in acute pancreatitis. We recommend the exclusive use of lactated Ringer’s solution as the primary resuscitation fluid in the early phase of acute pancreatitis.

## Figures and Tables

**Table 1:** Main characteristics of the included studies *: the abstracts published by Vasudevan and Reddy et al. refer to the same RCT, hence the total number of patients analyzed is 557. Abbreviations: LR: lactated Ringer’s solution; NS: normal saline; SD: standard deviation; IQR: interquartile range; SIRS: systemic inflammatory response syndrome; CRP: C-reactive protein; ESR: erythrocyte sedimentation rate; PCT: procalcitonin; LoH: length of hospitalization; OF: organ failure; HCO3: bicarbonate; ICU: need for intensive care; BUN: blood urea nitrogen; IL-6: interleukin-6.

Author, Year	Country	Data Source	Recruitment Period	Sample Size LR/NS	Age Mean ± SD/Median (IQR)	Males (%)	Baseline SIRS *n* (%)	Fluid Administration Protocol	Reported Outcomes
Choosakul, 2018 [43]	Thailand	full text	Nov 2015– Dec 2016	23/24	LR: 54.8 ± 20.4 NS: 48.3 ± 13.6	LR: 71 NS: 52	LR: 8 (35) NS: 10 (42)	Goal-directed; 20 mL/kg for 30 min, followed by 3 mL/kg/h; BUN controlled	SIRS, CRP, ESR, PCT, LoH, local compl., OF, mortality, severity, fluid infused 24h
de-Madaria, 2017 [44]	Spain	full text	Feb 2013– Mar 2015	19/21	LR: 63.8 ± 19.1 NS: 61.4 ± 15.5	LR: 52 NS: 42	LR: 9 (47) NS: 14 (67)	Goal-directed; 1000 mL 10% dextrose; 15 mL/kg for 60 min and 1.2 mL/kg/h or 10 mL/kg for 60 min and 1 mL/kg/h	SIRS, CRP, HCO3, pH, local compl., OF, severity, mortality, LoH, ICU, fluid infused 24h, nutritional support, invasive treatment
Farrell, 2018 [47]	USA	abstract	no data	38/38	LR: 12.1 ± 4.5 NS: 12.3 ± 5.3	LR: 43 NS: 50	LR: 6 (16) NS: 7 (18)	1.5 times maintenance fluid	SIRS, CRP, BUN, LoH, severity, local compl., time to full feeds
Karki, 2022 [30]	Nepal	full text	Oct 2018– Jun 2019	26/25	41.33 ± 14.17	LR: 96 NS: 4	LR: 12 (46) NS: 14 (64)	1000 mL 5% dextrose; 10 mL/kg for 1.5 mL/kg/h	SIRS, CRP, local compl., LoH, mortality, severity
Kayhan, 2021 [31]	Turkey	full text	Jan 2019– Sep 2019	67/65	LR: 54.6 ± 17.9 NS: 56.3 ± 17.2	LR: 52 NS: 48	NA	Goal-directed; 1000 mL in the first 60 min; 3 mL/kg/h	CRP, pH, HCO3, local compl., OF, severity, LoH
Lee, 2020 [45]	USA	full text	Sep 2018– Aug 2019	61/60	LR: 42.3 ± 14.0 NS: 43.5 ± 14.2	LR: 55 NS: 49	NA	10 mL/kg bolus; 3 mL/kg/h	SIRS, LoH, ICU, local compl., OF, severity, mortality, hyperchloremia, recurrence, need for intervention, fluid infused 24h, fluid before randomization
Reddy *, 2014 [42]	India	abstract	Jul 2012– Jun 2013	25/25	45.8 ± 16.5	56	NA	Goal-directed; 20 mL/kg bolus, individualized maintenance fluid	LoH, severity, IL-6, need for intervention, infective compl., OF, fluid infused 7 days
Vasudevan *, 2013 [46]	India	abstract	Jan 2012– Jun 2013	25/25	mean: 41.64	66	NA	Goal-directed; 20 mL/kg bolus, 3–5 mL/kg/h	LoH, local compl., ICU, OF, infective compl., need for intervention
Wu, 2011 [17]	USA	full text	May 2009– Feb 2010	19/21	LR: 50 (40–73) NS: 54 (40–60)	LR: 44 NS: 68	LR: 6 (32) NS: 4 (19)	Goal-directed and standard subgroups; 20 mL/kg bolus for 30 min, 3 mL/kg/h for 8–12 h, after either 20 mL/kg bolus and 3 mL/kg/h or no bolus and 1.5 mL/kg/h	SIRS, CRP, local compl., LoH, ICU, OF, mortality, infection, fluid before randomization

**Table 2:** Effect sizes, heterogeneity and quality of evidence for outcomes included in the meta-analyses. Abbreviations: AP: acute pancreatitis. Significant results are highlighted in bold.

Outcome	Studies	Patients	Overall Effect			Heterogeneity		GRADE	Importance
			RR/MD	95% CI	*p*	I^2^	*p*		
Moderate-to-severe AP	3	293	**0.59**	**0.36–0.97**	**0.045**	0%	0.692	⨁⨁⨁⨁ High	critical
Mortality	5	299	**0.48**	**0.24–0.98**	**0.047**	0%	0.979	⨁◯◯◯ Very low	critical
Length of hospitalization (days)	8	557	−0.57	−1.33–0.19	0.120	35.7%	0.100	⨁⨁⨁◯ Moderate	critical
Organ failure	6	430	**0.78**	**0.61–0.99**	**0.046**	0%	0.842	⨁⨁◯◯ Low	critical
Need for intensive care	4	251	**0.50**	**0.33–0.77**	**0.014**	0%	0.861	⨁⨁◯◯ Low	important but not critical
Local complications	4	351	**0.64**	**0.46–0.89**	**0.023**	0%	0.856	⨁⨁⨁◯ Moderate	critical
Necrosis	7	420	0.70	0.40–1.23	0.176	0%	0.618	⨁⨁◯◯ Low	critical
Pseudocyst	3	174	0.96	0.11–8.68	0.950	0%	0.659	⨁⨁◯◯ Low	important but not critical
SIRS at 24 h	6	374	0.77	0.33–1.82	0.473	32%	0.204	-	of limited importance
SIRS at 48 h	4	273	0.92	0.92–2.92	0.827	31%	0.224	-	of limited importance
SIRS at 72 h	3	212	0.69	0.35–1.33	0.135	0%	0.777	-	of limited importance
CRP at 48 h (mg/L)	3	232	−51.03	−231.90–129.84	0.350	85.9%	<0.1	-	of limited importance
Fluid administered 24 h	3	208	−152.12	−1024.68–720.45	0.530	10.3%	0.300	-	of limited importance

## Data Availability

The data presented in this study are available in the supplementary material.

## Supplementary Materials

The following supporting information can be downloaded at: https://www.mdpi.com/article/10.3390/biomedicines11020321/s1; Figure S1. Results of risk of bias assessment with RoB 2 tool; Figure S2. Moderate-to-severe acute pancreatitis; Figure S3. In-hospital mortality; Figure S4. Length of hospital stay; Figure S5. Need for intensive care; Figure S6. Organ failure; Figure S7. Local complications; Figure S8. Necrosis; Figure S9. Pseudocyst; Figure S10. Systemic inflammatory response syndrome; Figure S11. C-reactive protein levels (mg/dL) at 24, 48 and 72 h after randomization; Figure S12. Amount of fluid administered in the first 24 h after randomization; Table S1. Detailed results of GRADE assessment. Table S2. References of the included studies.
