# Supplementary material for: Lactated Ringer’s Solution Reduces Severity, Mortality, Systemic and Local Complications in Acute Pancreatitis: A Systematic Review and Meta-Analysis
Source: Biomedicines. 2023 Jan 23;11(2):321. doi: 10.3390/biomedicines11020321 (PMC9953296; doi:10.3390/biomedicines11020321)
Supplement: Supplementary file 1 [file biomedicines-11-00321-s001.zip › biomedicines-2114220-supplementary-Final.pdf]

	Outcome	D1	D2	D3	D4	D5	Overall
de-Madaria et al. (2017)	MSAP	+	+	+	+	+	+
Lee et al. (2021)	MSAP	+	+	+	+	+	+
Kayhan et al. (2021)	MSAP	+	!	+	!	!	!
Choosakul et al. (2018)	mortality	+	+	+	+	+	+
de-Madaria et al. (2017)	mortality	+	+	+	+	+	+
Karki et al. (2022)	mortality	+	+	+	+	+	+
Lee et al. (2021)	mortality	+	+	+	+	+	+
Wu et al. (2011)	mortality	+	+	+	+	+	+
Choosakul et al. (2018)	LoH	+	+	+	+	+	+
de-Madaria et al. (2017)	LoH	+	+	+	+	+	+
Karki et al. (2022)	LoH	+	+	+	+	+	+
Lee et al. (2021)	LoH	+	+	+	+	+	+
Wu et al. (2011)	LoH	+	+	+	+	+	+
Farrell et al. (2022)	LoH	+	+	+	+	+	+
Kayhan et al. (2021)	LoH	+	!	+	+	!	!
Reddy/vasudevan et al. (2014)	LoH	!	-	!	+	!	-
de-Madaria et al. (2017)	ICU	+	+	+	+	+	+
Lee et al. (2021)	ICU	+	+	+	+	+	+
Wu et al. (2011)	ICU	+	+	+	+	+	+
Reddy/Vasudevan et al. (2014)	ICU	!	-	+	!	!	-
Choosakul et al. (2018)	OF	+	+	+	+	+	+
de-Madaria et al. (2017)	OF	+	+	+	+	+	+
Lee et al. (2021)	OF	+	+	+	+	+	+
Wu et al. (2011)	OF	+	+	+	!	+	!
Kayhan et al. (2021)	OF	+	!	+	+	!	!
Reddy/Vasudevan et al. (2014)	OF	!	-	+	!	!	-
Choosakul et al. (2018)	Local compl.	+	+	+	+	+	+
Karki et al. (2022)	Local compl.	+	+	+	+	+	+
Lee et al. (2021)	Local compl.	+	+	+	+	+	+
Kayhan et al. (2021)	Local compl.	+	!	+	+	!	!
Choosakul et al. (2018)	Necrosis	+	+	+	+	+	+
de-Madaria et al. (2017)	Necrosis	+	+	+	+	+	+
Karki et al. (2022)	Necrosis	+	+	+	+	+	+
Wu et al. (2011)	Necrosis	+	+	+	+	+	+
Farrell et al. (2022)	Necrosis	+	+	+	+	+	+
Kayhan et al. (2021)	Necrosis	+	!	+	+	!	!
Reddy/vasudevan et al. (2014)	Necrosis	!	-	!	+	!	-
Choosakul et al. (2018)	Pseudocyst	+	+	+	+	+	+
Karki et al. (2022)	Pseudocyst	+	+	+	+	+	+
Farrell et al. (2022)	Pseudocyst	+	+	+	+	+	+
Choosakul et al. (2018)	CRP	+	+	+	-	+	-
de-Madaria et al. (2017)	CRP	+	+	+	+	+	+
Wu et al. (2011)	CRP	+	+	+	+	!	!
Farrell et al. (2022)	CRP	+	+	+	+	+	+
Kayhan et al. (2021)	CRP	+	!	+	+	!	!
Choosakul et al. (2018)	SIRS	+	+	+	+	+	+
de-Madaria et al. (2017)	SIRS	+	+	+	+	+	+
Karki et al. (2022)	SIRS	+	+	+	+	+	+

+	Low risk
!	Some concerns
-	High risk
D1	Randomisation process
D2	[illegible]

Figure S1. Results of risk of bias assessment with RoB2 tool. MSAP: moderate-to-severe acute pancreatitis; LoH: length of hospitalization; ICU: intensive care unite admission; OF: organ failure; CRP: C-reactive protein; SIRS: systemic inflammatory response syndrome.

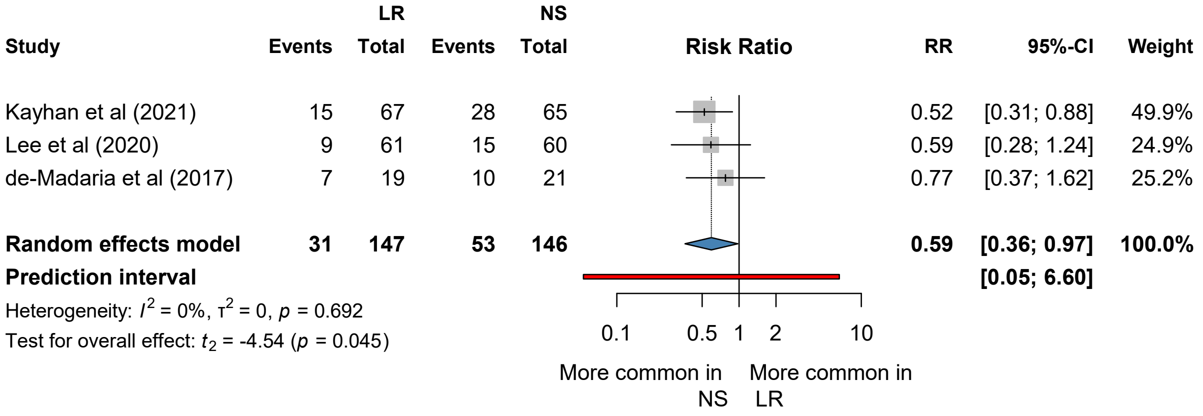

Figure S2. Moderate-to-severe acute pancreatitis. Statistical heterogeneity across trials was assessed by means of the Cochrane Q test and the I^2 values.

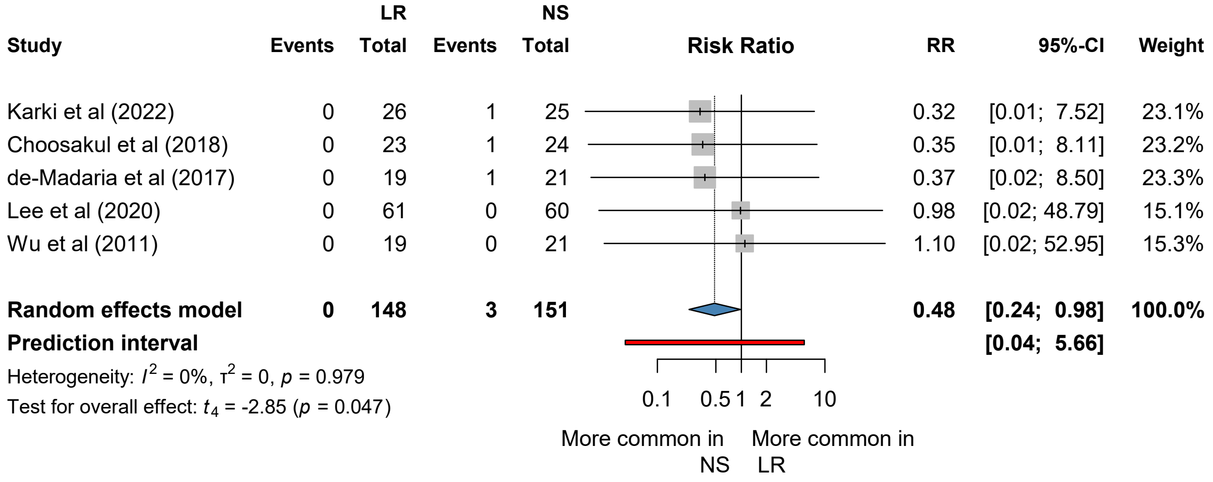

Figure S3. In-hospital mortality. Statistical heterogeneity across trials was assessed by means of the Cochrane Q test and the I^2 values.

Studies	Lactated Ringer's solution (LR) N	Median	Q1	Q3	Mean	SD	Normal saline (NS) N	Median	Q1	Q3	Mean	SD	Median difference	95% CI	Weight
Reddy/Vasudevan et al (2014)	25				14.0	7.2	25				22.0	12.4	-8.00	[-13.63; -2.37]	0.9%
Lee et al (2020)	61	3.5	2.0	5.9			60	4.6	3.0	7.4			-1.10	[-2.43; 0.23]	13.8%
Karki et al (2022)	26				5.2	0.1	25				6.2	2.5	-1.05	[-2.03; -0.07]	23.0%
Wu et al (2011)	19	5.0	3.0	6.0			21	5.5	5.0	8.0			-0.50	[-2.26; 1.26]	8.2%
Farrell et al (2022)	38	2.8	1.7	5.0			38	3.3	2.5	4.8			-0.50	[-1.66; 0.66]	17.5%
de-Madaria et al (2017)	19	9.0	5.8	13.0			21	9.0	5.0	16.0			0.00	[-5.10; 5.10]	1.0%
Kayhan et al (2021)	67	3.0	2.0	4.0			65	3.0	2.0	5.0			0.00	[-0.80; 0.80]	31.7%
Choosakul et al (2018)	23	6.0	4.0	9.0			24	5.5	4.0	9.0			0.50	[-2.11; 3.11]	3.9%
**Random effects model**													**-0.57**	**[-1.33; 0.19]**	**100.0%**
**Prediction interval**														**[-1.45; 0.31]**	

Heterogeneity: I^2 = 35.7% [0.0%; 71.6%], τ^2 = 0.0591, p = 0.1
Test for overall effect: t_7 = -1.79 (p = 0.12)

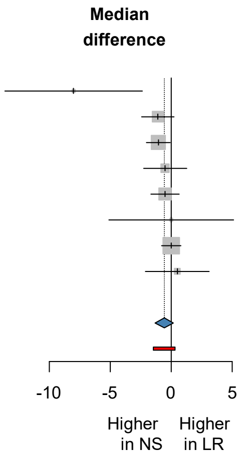

Figure S4. Length of hospital stay. Statistical heterogeneity across trials was assessed by means of the Cochrane Q test and the I² values.

Study	LR Events	LR Total	NS Events	NS Total	RR	95%-CI	Weight
de-Madaria et al (2017)	0	19	1	21	0.37	[0.02; 8.50]	2.7%
Wu et al (2011)	1	19	3	21	0.37	[0.04; 3.25]	5.6%
Lee et al (2020)	6	61	15	60	0.39	[0.16; 0.95]	34.7%
Reddy/Vasudevan et al (2014)	8	25	13	25	0.62	[0.31; 1.22]	57.0%
**Random effects model**	**15**	**124**	**32**	**127**	**0.50**	**[0.33; 0.77]**	**100.0%**
**Prediction interval**						**[0.16; 1.57]**	

Heterogeneity: I^2 = 0%, τ^2 = 0, p = 0.861
Test for overall effect: t_3 = -5.18 (p = 0.014)

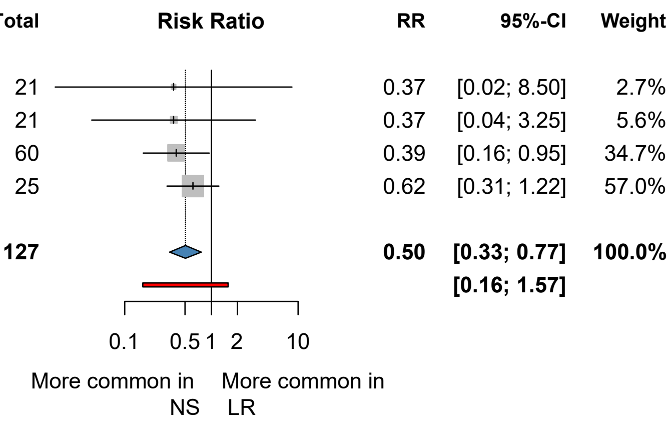

Figure S5. Need for intensive care. Statistical heterogeneity across trials was assessed by means of the Cochrane Q test and the I² values.

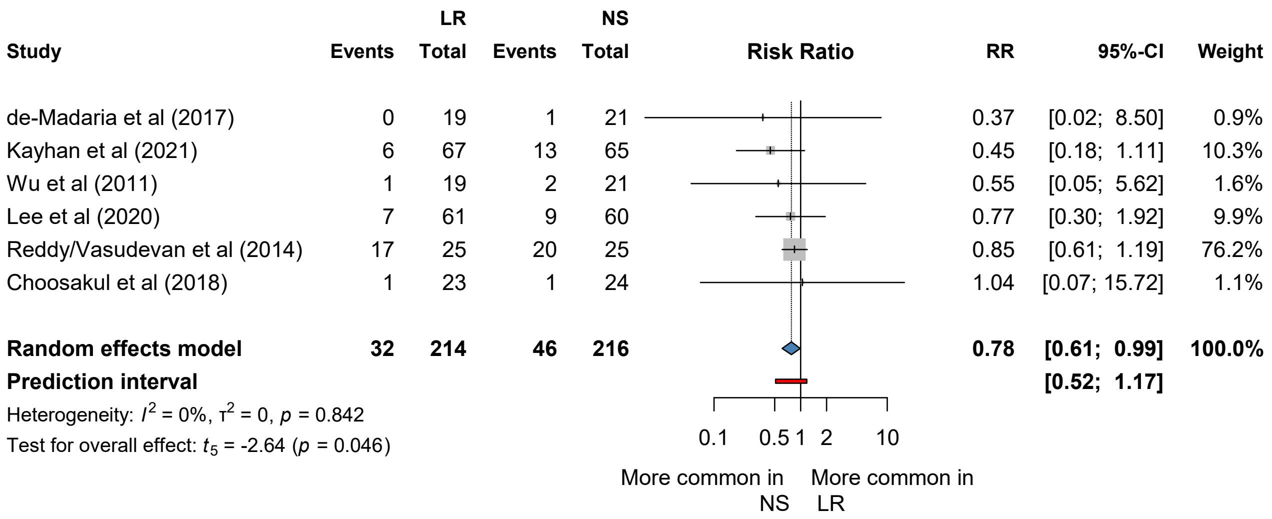

Figure S6. Organ failure. Statistical heterogeneity across trials was assessed by means of the Cochrane Q test and the I² values.

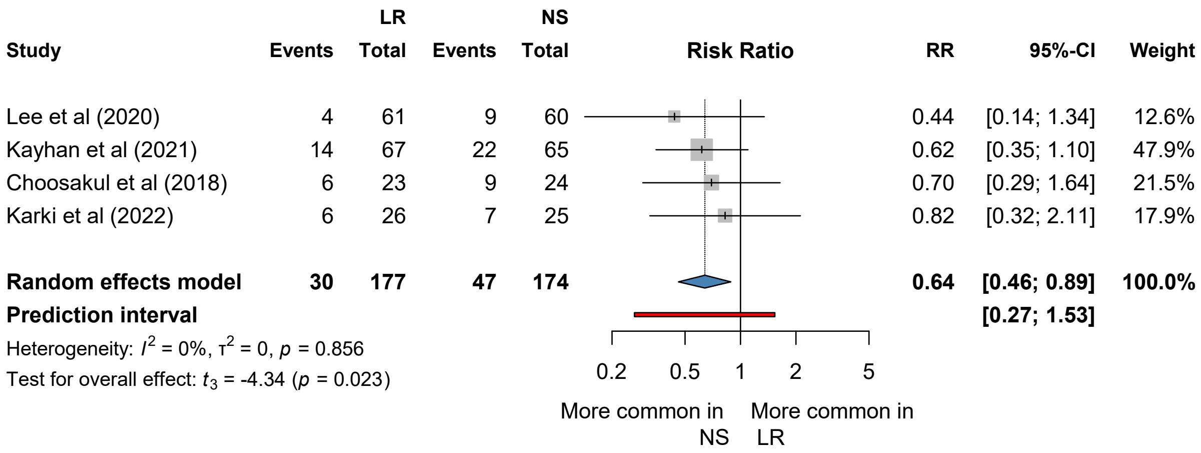

Figure S7. Local complications. Statistical heterogeneity across trials was assessed by means of the Cochrane Q test and the I² values.

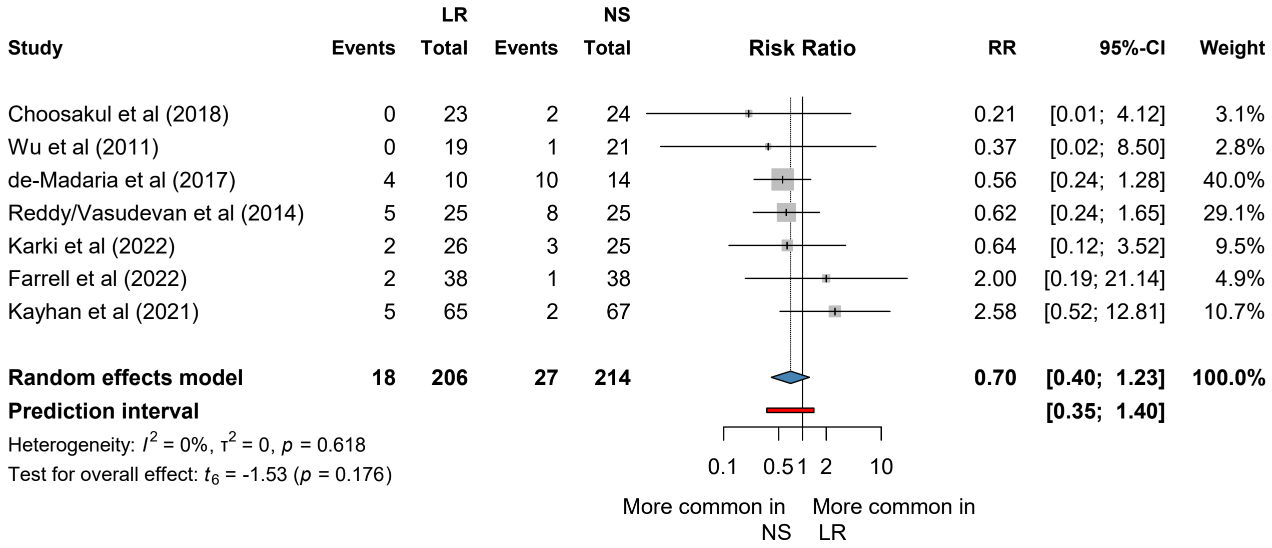

Figure S8. Necrosis. Statistical heterogeneity across trials was assessed by means of the Cochrane Q test and the I² values.

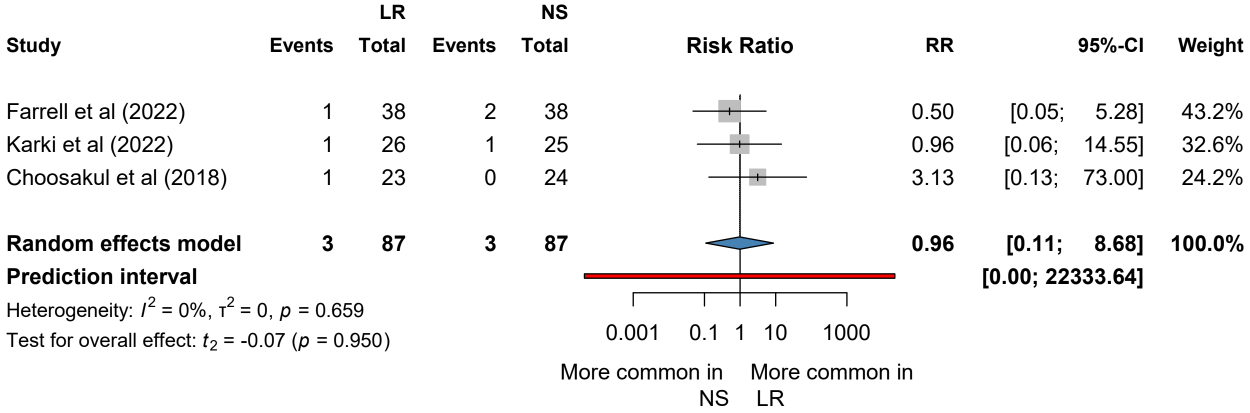

Figure S9. Pseudocyst. Statistical heterogeneity across trials was assessed by means of the Cochrane Q test and the I² values.

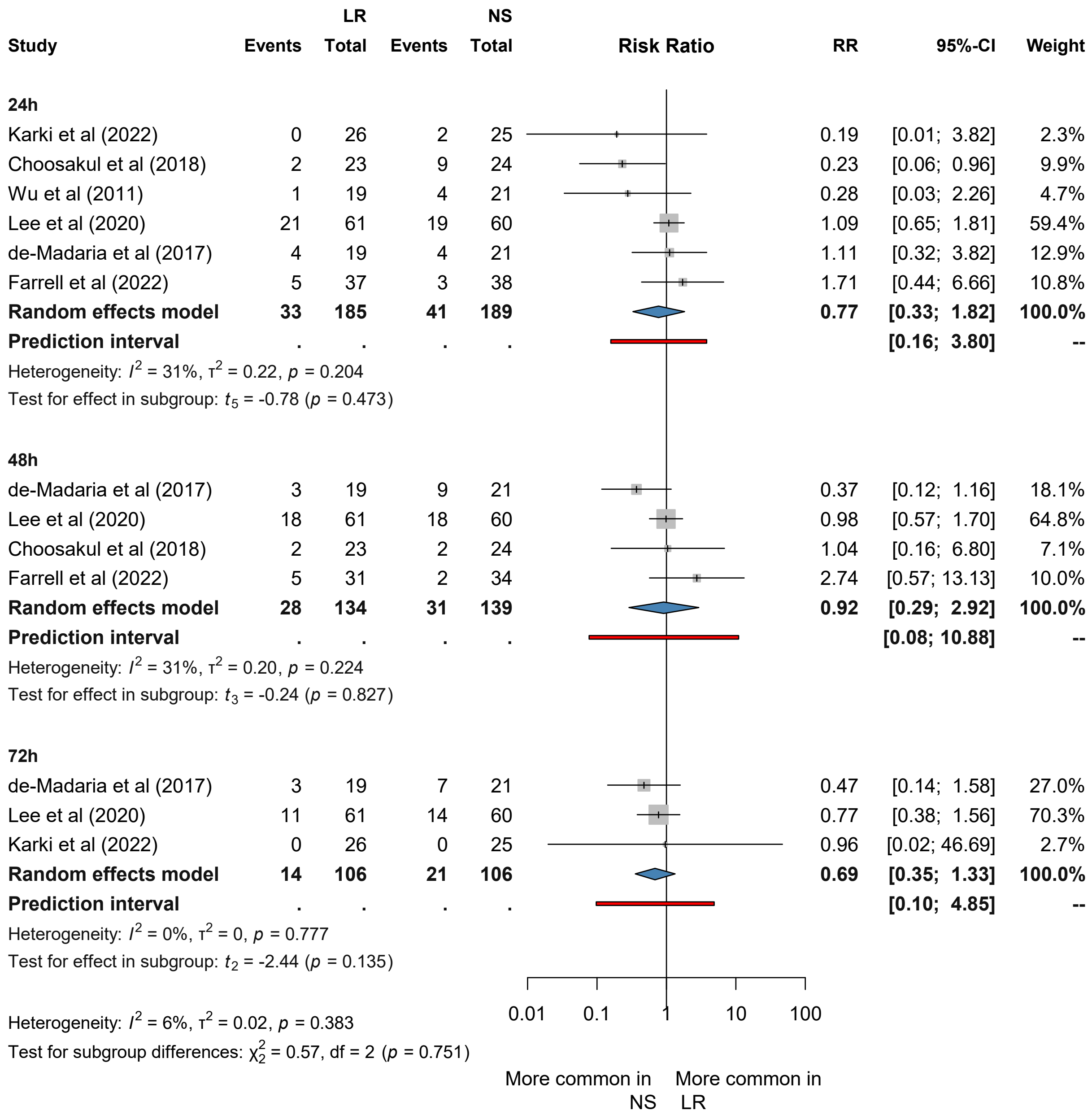

Figure S10. Systemic inflammatory response syndrome. Statistical heterogeneity across trials was assessed by means of the Cochrane Q test and the I² values.

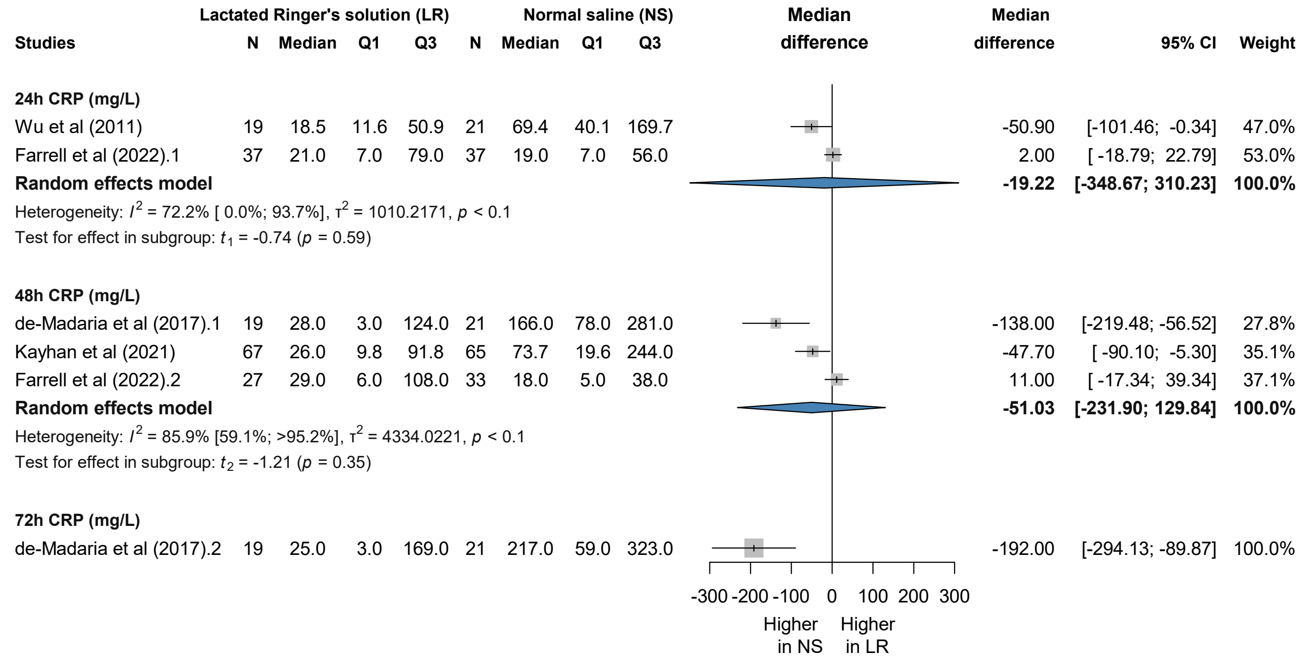

Figure S11. C-reactive protein levels (mg/dL) at 24, 48 and 72 h after randomization. Statistical heterogeneity across trials was assessed by means of the Cochrane Q test and the I² values.

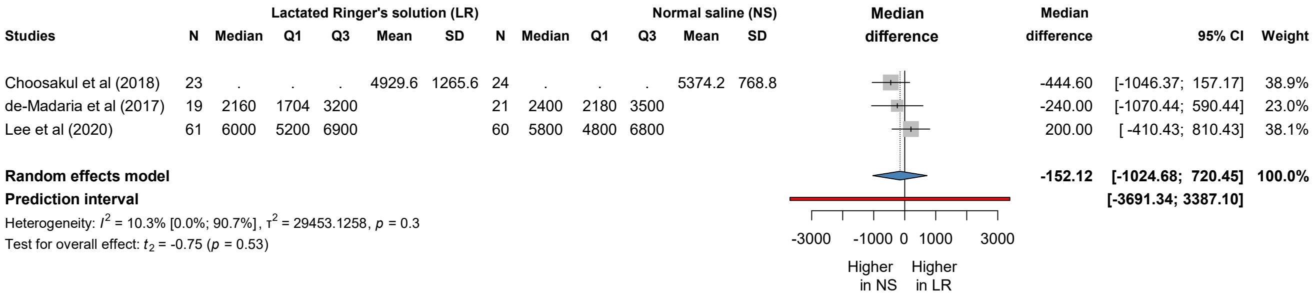

Figure S12. Amount of fluid administered in the first 24 h after randomization. Statistical heterogeneity across trials was assessed by means of the Cochrane Q test and the I² values.

Table S1. Detailed results of GRADE assessment.

Certainty Assessment							№ of Patients		Effect			
№ of Studies	Study Design	Risk of Bias	Inconsistency	Indirectness	Imprecision	Other considerations	Lactated Inger's Solution	Normal Saline	Relative (95% CI)	Absolute (95% CI)	Certainty	Importance
**Moderate-to-severe AP (assessed with: Atlanta classification)**												
3	RCT	not serious	not serious	not serious	not serious[a]	none	31/147 (21.1%)	53/146 (36.3%)	**RR 0.59** (0.36 to 0.97)	**149 fewer per 1 000** (from 232 fewer to 11 fewer)	⊕⊕⊕⊕ High	**critical**
**In-hospital mortality**												
5	RCT	not serious	not serious	not serious	extremely serious[b]	none	0/148 (0.0%)	3/151 (2.0%)	**RR 0.48** (0.24 to 0.98)	**10 fewer per 1 000** (from 15 fewer to 0 fewer)	⊕◯◯◯ Very low	**critical**
**Length of hospitalization (assessed with: days)**												
8	RCT	serious[c]	not serious[d]	not serious	not serious	none	278	279	-	MD **0.57 days more** (1.33 fewer to 0.19 more)	⊕⊕⊕◯ Moderate	**critical**
**Need for intensive care**												
4	RCT	serious[e]	not serious	not serious	serious[f]	none	15/124 (12.1%)	32/127 (25.2%)	**RR 0.50** (0.33 to 0.77)	**126 fewer per 1 000** (from 169 fewer to 58 fewer)	⊕⊕◯◯ Low	**important but not critical**
**Organ failure**												

6	RCT	very serious[c]	not serious	not serious	not serious	none	32/214 (15.0%)	46/216 (21.3%)	**RR 0.78** (0.61 to 0.99)	**47 fewer per 1 000** (from 83 fewer to 2 fewer)	⊕⊕◯◯ Low	**critical**
**Local complications**												
4	RCT	not serious[g]	not serious	not serious	serious[h]	none	30/177 (16.9%)	47/174 (27.0%)	**RR 0.64** (0.46 to 0.89)	**97 fewer per 1 000** (from 146 fewer to 30 fewer)	⊕⊕⊕◯ Moderate	**critical**
**Necrosis**												
7	RCT	very serious[i]	not serious	not serious	not serious	none	16/206 (7.8%)	28/214 (13.1%)	**RR 0.70** (0.40 to 1.23)	**39 fewer per 1 000** (from 79 fewer to 30 more)	⊕⊕◯◯ Low	**critical**
**Pseudocyst**												
3	RCT	not serious[i]	not serious	not serious	very serious[f]	none	3/87 (3.4%)	3/87 (3.4%)	**RR 0.96** (0.11 to 8.68)	**1 fewer per 1 000** (from 31 fewer to 265 more)	⊕⊕◯◯ Low	**important but not critical**

a. In our cohort, moderate plus severe cases were 29%; calculating with a 50% reduction, OIS is 121 per group.

b. Mortality rate is under 5%, therefore, considering OIS more than 500.

c. One high risk and one some concerns study.

d. Heterogeneity originates from Reddy (has a small sample size, not influential).

e. Reddy/ Vasudevan with high risk of bias produced most patients with event.

f. Considering the number of patients with event, total number of patients in analysis does not reach OIS.

g. Half of the data comes from study with some concerns.

h. A 13% incidence of local compl., OIS is not reached.

i. Proportion of studies carrying high risk of bias or some concern is high.

Table S2. References of the included studies (as per the reference list of the manuscript).

Reference Number	Reference
17.	Wu, B.U.; Hwang, J.Q.; Gardner, T.H.; Repas, K.; Delee, R.; Yu, S.; Smith, B.; Banks, P.A.; Conwell, D.L. Lactated Ringer's Solution Reduces Systemic Inflammation Compared with Saline in Patients with Acute Pancreatitis. Clin. Gastroenterol. Hepatol. 2011, 9, P710–P717.e1. https://doi.org/10.1016/j.cgh.2011.04.026.
30.	Karki, B.; Thapa, S.; Khadka, D.; Karki, S.; Shrestha, R.; Khanal, A.; Shrestha, R.; Paudel, B.N. Intravenous Ringers lactate versus normal saline for predominantly mild acute pancreatitis in a Nepalese Tertiary Hospital. PLoS ONE 2022, 17, e0263221. https://doi.org/10.1371/journal.pone.0263221.
31.	Kayhan, S.; Akyol, B.S.; Ergul, M.; Baysan, C. The effect of type of fluid on disease severity in acute pancreatitis treatment. Eur. Rev. Med. Pharmacol. Sci. 2021, 25, 7460–7467.
42.	Reddy, Y.R.; Talukder, S.; Yadav, T.D.; Siddappa, P.K.; Kochhar, R. Effect of intravenous fluid resuscitation on inflammatory markers of acute pancreatitis and its clinical outcome. United Eur. Gastroenterol. J. 2014, 2, 132–135. https://doi.org/10.1177/2050640614548980.
43.	Choosakul, S.; Harinwan, K.; Chirapongsathorn, S.; Opuchar, K.; Sanpajit, T.; Piyanirun, W.; Puttapitakpong, C. Comparison of normal saline versus Lactated Ringer's solution for fluid resuscitation in patients with mild acute pancreatitis, A randomized controlled trial. Pancreatology 2018, 18, 507–512. https://doi.org/10.1016/j.pan.2018.04.016.
44.	de-Madaria, E.; Herrera-Marante, I.; Gonzalez-Camacho, V.; Bonjoch, L.; Quesada-Vazquez, N.; Almenta-Saavedra, I.; Miralles-Macia, C.; Acevedo-Piedra, N.G.; Roger-Ibanez, M.; Sanchez-Marin, C.; et al. Fluid resuscitation with lactated Ringer's solution vs normal saline in acute pancreatitis: A triple-blind, randomized, controlled trial. United Eur. Gastroenterol. J. 2018, 6, 63–72. https://doi.org/10.1177/2050640617707864.
45.	Lee, A.; Ko, C.; Buitrago, C.; Hiramoto, B.; Hilson, L.; Buxbaum, J.; Grp, N.-L.S. Lactated Ringers vs Normal Saline Resuscitation for Mild Acute Pancreatitis: A Randomized Trial. Gastroenterology 2021, 160, 955. https://doi.org/10.1053/j.gastro.2020.10.044.
46.	Vasu De Van, P.; Verma, G.R.; Bhalla, A.; Kalra, N.; Basha, J.; Appasani, S.; Chhabra, P.; Manrai, M.; Sinha, S.K.; Singh, K.; et al. Does the type of fluid used in resuscitation matter in the clinical course of acute pancreatitis? Indian J. Gastroenterol. 2013, 32, A110. https://doi.org/10.1007/s12664-013-0417-z.
47.	Farrell, P.R.; DesPain, A.W.; Farmer, P.F.; Farrell, L.M.; Greenfield, B.; Rogers, M.; Hornung, L.; Kim, E.; Pearman, R.; Neway, B.; et al. Mitigating the Inflammatory Response in Acute Pancreatitis; A Randomized Clinical Control Trial Comparing the Effects of Lactated Ringers and Normal Saline. Gastroenterology 2022, 162, S140–S141.
